# Prenatal Betamethasone interferes with immune system development and alters target cells in autoimmune diabetes

**DOI:** 10.1038/s41598-018-37878-9

**Published:** 2019-02-04

**Authors:** David Perna-Barrull, Silvia Rodriguez-Fernandez, Irma Pujol-Autonell, Anna Gieras, Rosa M. Ampudia-Carrasco, Adrian Villalba, Laura Glau, Eva Tolosa, Marta Vives-Pi

**Affiliations:** 1 Immunology Section, Germans Trias i Pujol Research Institute, Autonomous University of Barcelona, Badalona, Spain; 2 Department of Immunology, University Medical Center Hamburg-Eppendorf, Hamburg, Germany; 3 CIBERDEM, Barcelona, Spain

## Abstract

Non-genetic factors are crucial in the pathogenesis of type 1 diabetes (T1D), a disease caused by autoimmunity against insulin-producing β-cells. Exposure to medications in the prenatal period may influence the immune system maturation, thus altering self-tolerance. Prenatal administration of betamethasone –a synthetic glucocorticoid given to women at risk of preterm delivery– may affect the development of T1D. It has been previously demonstrated that prenatal betamethasone administration protects offspring from T1D development in nonobese diabetic (NOD) mice. The direct effect of betamethasone on the immature and mature immune system of NOD mice and on target β-cells is analysed in this paper. *In vitro*, betamethasone decreased lymphocyte viability and induced maturation-resistant dendritic cells, which in turn impaired γδ T cell proliferation and decreased IL-17 production. Prenatal betamethasone exposure caused thymus hypotrophy in newborn mice as well as alterations in immune cells subsets. Furthermore, betamethasone decreased β-cell growth, reduced C-peptide secretion and altered the expression of genes related to autoimmunity, metabolism and islet mass in T1D target tissue. These results support the protection against T1D in the betamethasone-treated offspring and demonstrate that this drug alters the developing immune system and β-cells. Understanding how betamethasone generates self-tolerance could have potential clinical relevance in T1D.

## Introduction

Type 1 diabetes mellitus (T1D) is a metabolic disease caused by the destruction of pancreatic β-cells by autoreactive T lymphocytes^1^. T1D is usually diagnosed during childhood or adolescence, and its incidence is steadily increasing an average of 4% per year^2^. Many autoimmune diseases display a similar increase in incidence, but so far, there is no explanation for this trend. The challenge in finding a cure is based on the heterogeneity of mechanisms that ultimately lead to the development of autoimmune reactions. For this reason, a better understanding of the influence of non-genetic factors in T1D susceptibility is needed.

Predisposition to this disease relies mainly on genetic and environmental factors. Changes in the environment in the last decades have critically contributed to the increase in T1D incidence^3^ and other autoimmune diseases. Recently, special attention has been focused on pre- and perinatal factors. An indirect evidence of their contribution in this matter is that dizygotic twins show greater incidence of T1D (15–25%)^4,5^ than non-twin siblings (5–10%)^4,6^. The age of the mother is also relevant to the increasing incidence of T1D^7^. Moreover, the greater number of alloreactive microchimeric maternal cells in autoimmune diseases^8^ and specifically in the pancreas from patients with T1D can be related to the pathogenesis of the disease^9^. More recently, a preprimed CD4^+^ T cell population with capacity to respond immunologically to β-cell antigens was found in newborns, and these cells seem to be generated by an inefficient antigen presentation in the first months of life^10^.

Considering the pivotal relevance of the prenatal time window and how extrinsic factors can determine the development of autoimmunity/T1D in later life, the use of medical interventions during this period can alter the immune response in adult life. In a spontaneous T1D experimental model, the non-obese diabetic (NOD) mouse, we have recently shown that prenatal administration of betamethasone reduced T1D incidence in the offspring and changed the TCR repertoire^11^. Betamethasone –a synthetic glucocorticoid widely used in obstetrics– induced foetal lung maturation, and significantly reduced mortality and morbidity in preterm newborns^12^. This drug easily reaches the foetus because it is not affected by inactivation by the placental enzyme 11β-hydroxysteroid dehydrogenase 2^13^. Moreover, betamethasone also targets the ubiquitous glucocorticoid receptor, which is present in almost all cell types, including leukocytes, and may therefore affect the development of the immune system^13^.

One of the effects of betamethasone on T lymphocytes is the alteration of the T cell-receptor (TCR) repertoire. It has been previously demonstrated that prenatal betamethasone treatment results in a biased TCR repertoire, affecting the TCR (Vβ) relevant for T1D antigen recognition. In this scenario, the lower frequency of pathogenic TCRs (Vβ12 in CD4^+^ T cells and Vβ6 in CD8^+^ T cells) was reflected in the reduced immune cell infiltration in the pancreas and contributed to protection from T1D in the NOD mice^11^. These results encouraged us to analyse in depth the effects of prenatal betamethasone.

The effects of betamethasone on the immune system in NOD mice and in the insulin-producing β-cells, the target cells of T1D, are reported in this study. The results provide an explanation for the reduced incidence of T1D in NOD offspring after prenatal betamethasone treatment, enlightening possible immunological mechanisms, and adding knowledge in the complex puzzle that shapes the development of the immune system.

## Results

### Betamethasone deteriorates splenocytes’ viability

**Figure 1:**
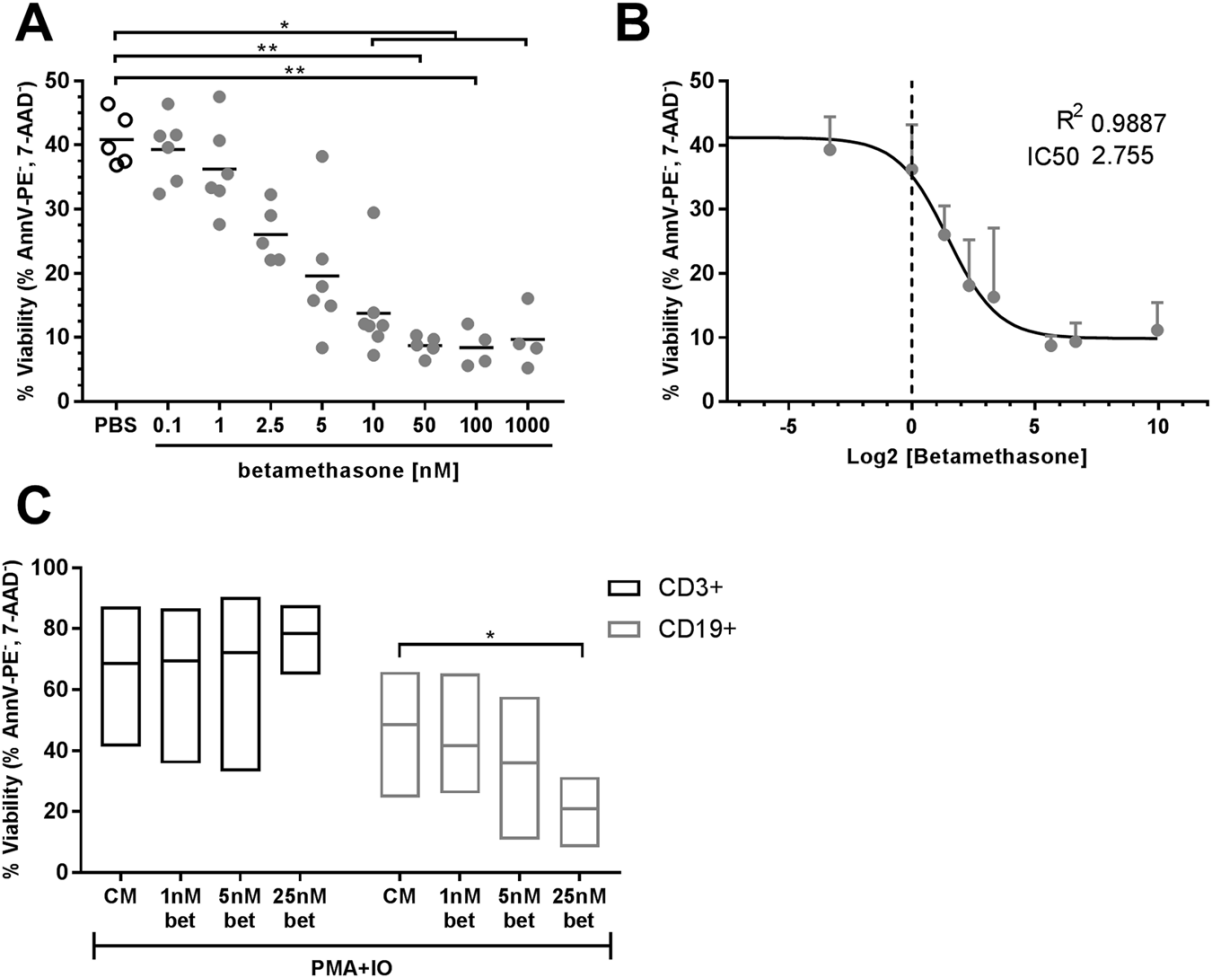
Betamethasone affects splenocytes’ viability and proliferation *in vitro*. (**A**) Percentage of viable splenocytes (annexinV PE^−^, 7aad^−^) after 24 h of culture with increasing betamethasone concentrations [0.1; 1; 2.5; 5; 10; 50; 100 and 1000 nM] (grey dots), and control condition (white circles). Lines show the mean of n ≥ 4 independent experiments (*p ≤ 0.05 and **p < 0.01, Dunn’s test, Kruskal-Wallis). (**B**) Sigmoidal non-linear regression of the splenocytes’ viability and the Log2 of the betamethasone concentration to determine the inhibitory concentration 50 (R2 = 0.9887 and IC50 = 2.755 nM). (**C**) Percentage of viable T cells (annexinV-PE^−^, 7aad^−^ of CD3^+^ splenocytes) and B cells (annexinV-PE^−^, 7aad^−^ of CD19^+^ splenocytes) after 72 h of culture with betamethasone (bet). White bars correspond to T cells with phorbol 12-miristate 13-acetate and ionomicine (PMA + IO) cultured with complete media (CM), 1 nM, 5 nM or 25 nM bet. Grey bars correspond to B cells with PMA + IO cultured with CM, 1 nM, 5 nM or 25 nM bet. Results presented as range and mean of 5 independent experiments (*p ≤ 0.05, Tukey’s test, Two-Way Anova).

Treatment with betamethasone causes apoptosis of immature thymocytes^11,14^. The direct effect of betamethasone on splenocytes was evaluated in this study. A dose-dependent reduction of splenocytes’ viability was observed after 24 h of culture, with a significant decrease starting at 10 nM betamethasone (Fig. 1A), reaching a plateau at 50 nM. Sigmoidal non-linear regression of the toxicity assay showed an inhibitory concentration 50 (IC50) of 2.755 nM betamethasone with an adjustment of the sigmoidal regression (R^2^) of 0.9887 (Fig. 1B). Moreover, after Phorbol 12-Myristate 13-Acetate (PMA) and ionomycin stimulation for 72 h, splenocytes viability was determined (Fig. 1C). No effect was observed in T lymphocytes viability. In contrast, a reduction in the viability of B cells was observed when 25 nM betamethasone was used.

To assess if this drug affects human leukocytes viability, we tested the *in vitro* effect of betamethasone in human peripheral blood mononuclear cells (PBMCs) from healthy donors. The results show that betamethasone affects leukocyte viability *in vitro* starting at 10^5^ nM (Suppl. Fig. 1). The effect was due to the decrease in viability of monocytes (CD14^+^) and B cells (CD19^+^).

In summary, betamethasone has a toxic effect on murine lymphocytes, and in human leukocytes, but at higher concentrations.

### Betamethasone treatment prevents full maturation of dendritic cells (DCs)

**Figure 2:**
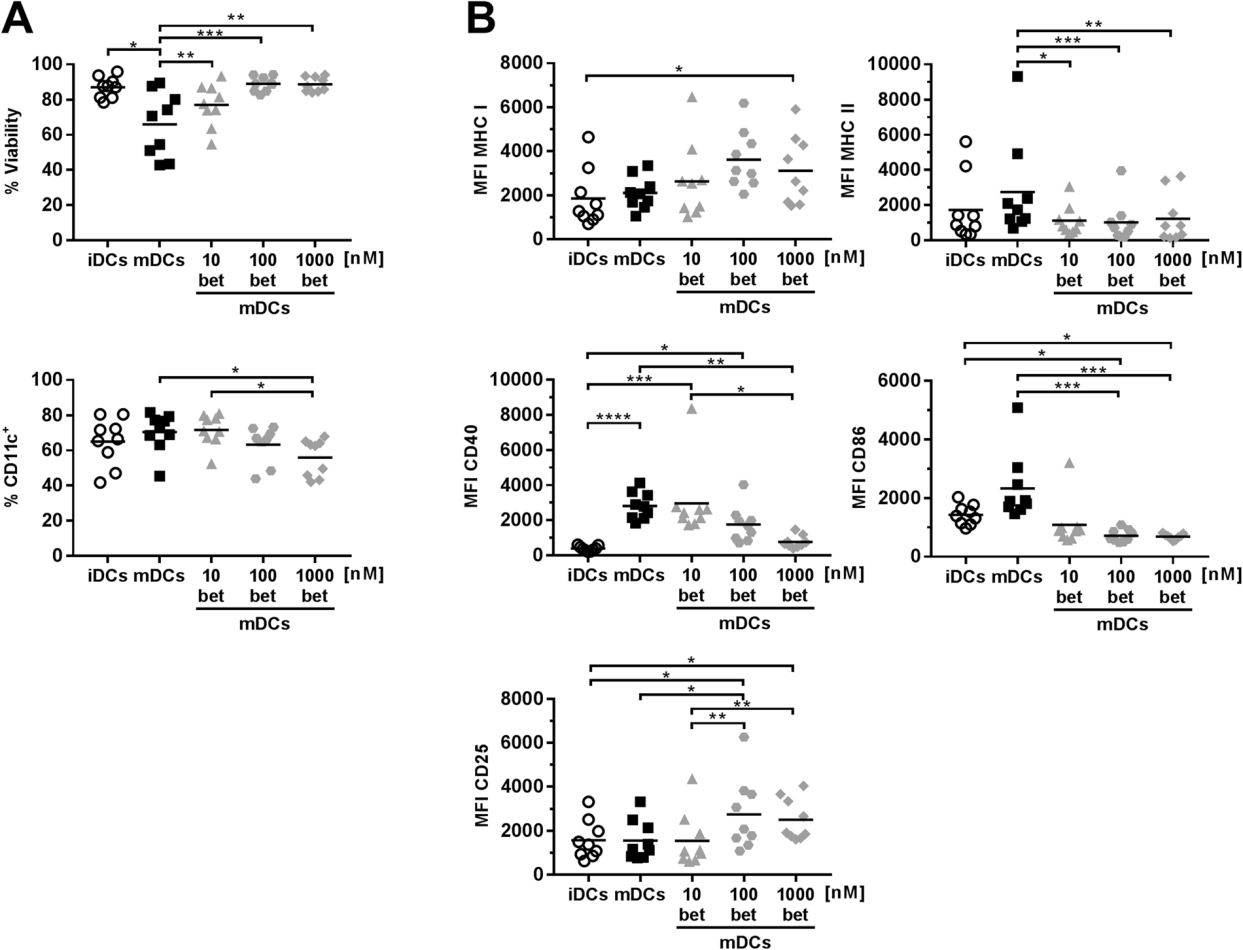
Dendritic cells (DCs) derived from bone marrow precursors in the presence of betamethasone show a semi-mature phenotype after LPS stimuli. (**A**) Upper panel: percentage of viability of DCs (annexinV PE^−^, 7aad^−^ of CD11c^hi^). Lower panel: differentiation yield of DCs from bone marrow progenitors (% CD11c^+^). (**B**) Median of fluorescence intensity (MFI) of MHC class I, MHC class II, CD40, CD86 and CD25 surface expression on DCs (CD11c^+^). White circles represent immature DCs (iDCs) after differentiation. Black and grey symbols represent DCs stimulated with lipopolysaccharide (LPS) for 24 h, without betamethasone (bet) (mDCs, black squares), or with 10 nM bet (grey triangles), 100 nM bet (grey dots), 1000 nM bet (grey rhombus). Lines show the mean of 9 independent experiments (*p ≤ 0.05, **p < 0.01, ***p < 0.001, ****p < 0.0001, Dunn’s test, Friedman test).

Antigen presenting cells are crucial for the outcome of adaptive immune responses, either orchestrating an inflammatory or a tolerogenic response. To assess the effect of betamethasone on DCs maturation, bone marrow precursor cells were cultured with increasing betamethasone concentrations during the differentiation process. The viability and phenotype were assessed after maturation with lipopolysaccharide (LPS). Betamethasone improved DCs viability after LPS maturation in comparison to untreated mature DCs (mDCs) (Fig. 2A, upper panel), but decreased the percentage of CD11c^+^ cells (Fig. 2A lower panel). Surface expression of MHC class I, MHC class II, CD40, CD86 and CD25 was determined by flow cytometry (Fig. 2B). Betamethasone treatment did not alter MHC class I expression in mDCs. In contrast, a significant reduction in MHC class II expression was found in all the betamethasone mDCs (betDCs) conditions when compared to mDCs. CD40 expression was downmodulated in the 1000betDCs condition when compared to mDCs, reaching levels of immature DCs (iDCs). Regarding the CD86 expression, a significant downmodulation in 100betDCs and 1000betDCs conditions was also observed. Finally, the expression of CD25 –a marker of immunoregulation^15^– was upregulated in the 100betDCs when compared to mDCs. In summary, these data show that betamethasone prevents full maturation of DCs.

To validate the effect of betamethasone in DCs matured with other innate immune ligands, CpG was used as maturation stimuli. The results show that betamethasone affects similarly both DCs matured with LPS or CpG in terms of viability, yield, and phenotype (Suppl. Fig. 2), and that it still points to a semi-mature or tolerogenic phenotype. Taken together, these results demonstrate that both maturation stimuli are not as powerful in the presence of betamethasone.

### Betamethasone-generated DCs impaired proliferation of γδ^+^ T lymphocytes and decreased IL-17 production

**Figure 3:**
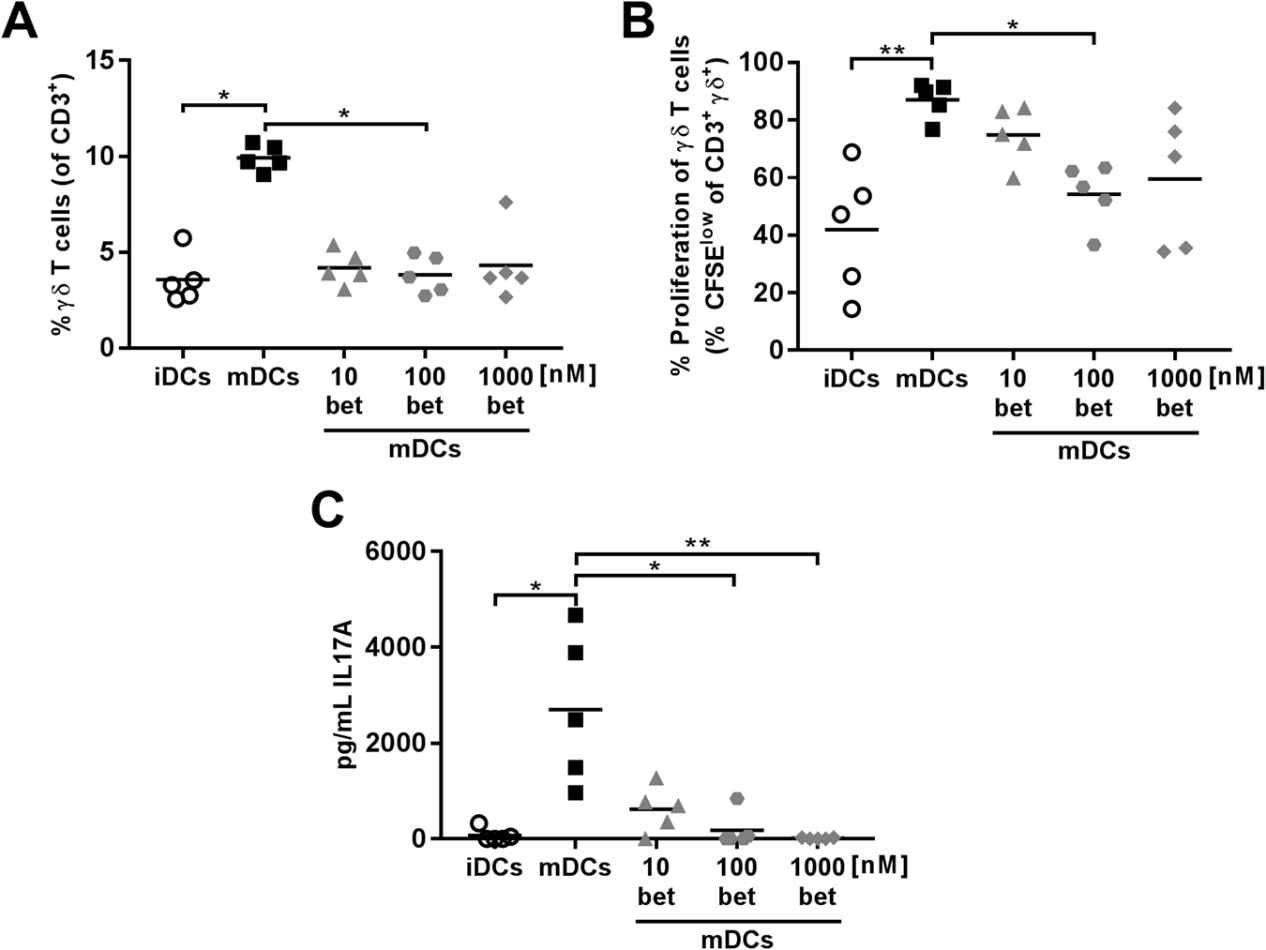
Dendritic cells (DCs) differentiated with betamethasone impair autologous γδ^+^ T lymphocyte proliferation. DCs were loaded with insulin (20 µg/ml) and cultured with splenocytes in a ratio 1:10 for 4 days. White circles represent immature DCs (iDCs), black squares represent mature DCs (mDCs) generated in basal conditions and stimulated with lipopolysaccharide (LPS), grey symbols represent mDCs with 10 nM (triangles), 100 nM (dots) and 1000 nM (rhombus) betamethasone (bet) during differentiation and stimulated with LPS. (**A**) Percentage of γδ^+^ T cells within CD3^+^ population after co-culture with DCs. (**B**) Percentage of autologous proliferation of γδ^+^ T cells (% CFSE^low^) after co-culture with DCs. (**C**) IL-17A production in T cell proliferation experiments measured in supernatants after 4 days of co-culture of DCs and splenocytes. Lines show the mean of 5 independent experiments (*p ≤ 0.05, **p < 0.01, Dunn’s test, Kruskal-Wallis test).

Since betamethasone inhibits DCs maturation, the ability of these DCs to induce lymphocyte proliferation was analysed. To that end, DCs exposed to betamethasone and LPS or CpG were co-cultured with Carboxyflourescein Diacetate Succinimidyl Ester (CFSE) stained splenocytes from NOD mice. Although no differences were found in the percentage of B lymphocytes, the percentage of CD3^+^ T lymphocytes was lower when DCs were exposed to betamethasone (1000betDCs) when compared to mDCs (Suppl. Fig. 3A). No differences were found in T and B lymphocyte proliferation induced by DCs (Suppl. Fig. 3A). Regarding DCs matured with CpG, we observed a similar T cell proliferation pattern in comparison to those matured with LPS (Suppl. Fig. 3B). Surprisingly, the percentage of γδ^+^ double negative (DN) CD3^+^ T cells was lower in 100betDCs condition when compared to mDCs condition (Fig. 3A and Suppl. Fig. 3B), even though the same trend was observed at concentrations of 10 and 1000 nM. Moreover, a significant inhibition of γδ^+^ DN CD3^+^ T cell proliferation was observed at 100 nM (Fig. 3B), demonstrating the biological effect of betamethasone. The secretion of IL-17 was determined in the supernatant of the co-culture of splenocytes with DCs. The results show a decrease in IL-17 secretion by betamethasone effect in proliferation experiments (Fig. 3C). In summary, proliferation of γδ^+^ T cells, but not of CD4^+^ and CD8^+^ T cells, is impaired after exposure to DCs generated with betamethasone and this effect is accompanied by a reduction of IL-17.

### Prenatal betamethasone causes thymus hypotrophy and alters the peripheral immune compartment in newborn NOD mice

**Figure 4:**
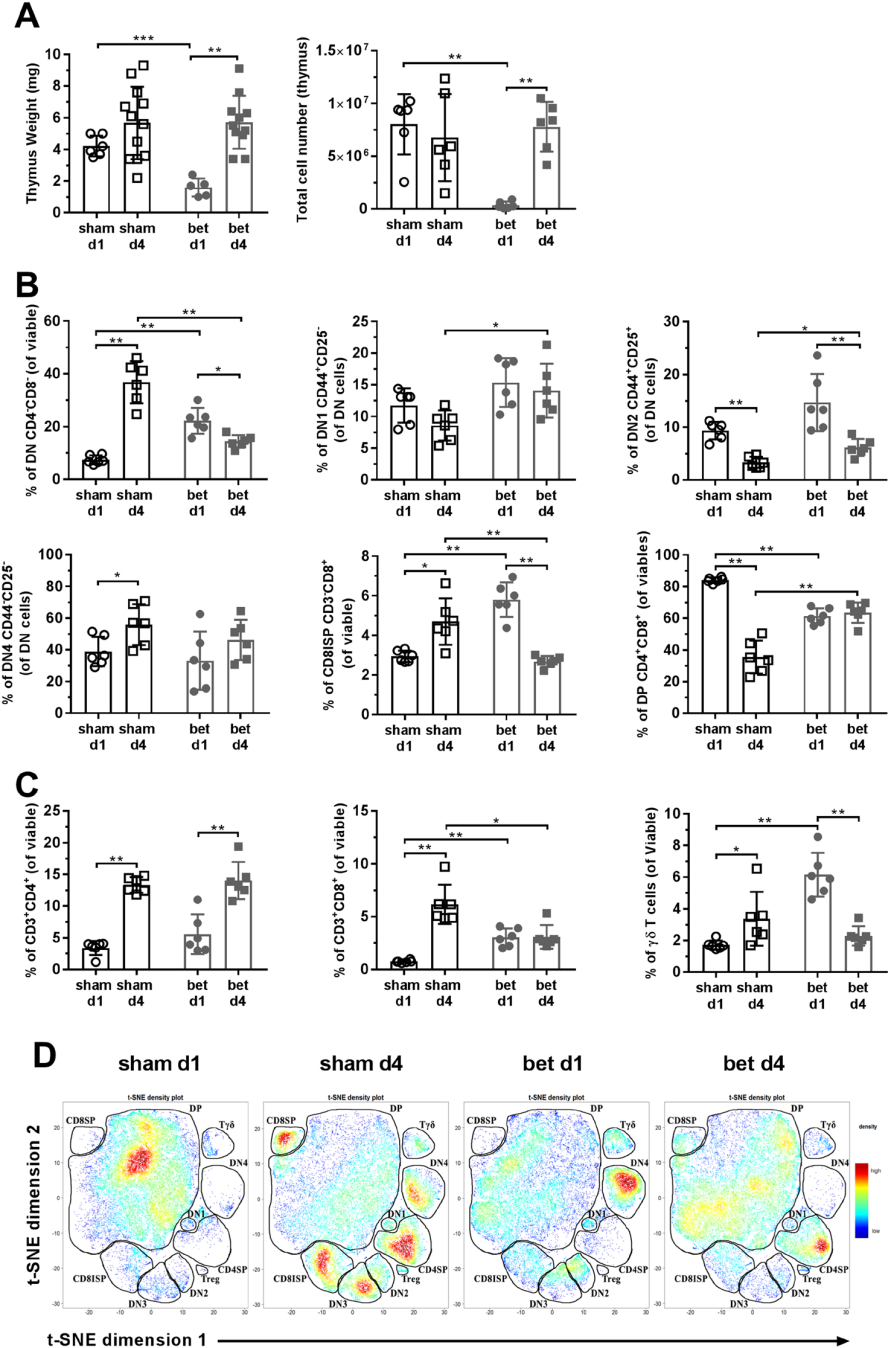
Prenatal betamethasone alters thymocyte maturation in the thymus. (**A**) Weight and total number of cells in the thymus. White symbols represent the control group and grey symbols represent the betamethasone (bet) group, circles or dots and squares were used for postnatal day 1 (d1) and postnatal day 4 (d4) after birth, respectively. Bars and symbols show the mean ± s.d. of n ≥ 5 mice (*p ≤ 0.05, **p < 0.01, ***p < 0.001, Mann-Whitney test). (**B**) Percentage of maturing thymocytes in the double negative (DN) stage (CD4^−^ CD8^−^), DN stage 1 (DN1, CD44^+^ CD25^−^), DN stage 2 (DN2, CD44^+^ CD25^+^), DN stage 4 (DN4, CD44^−^ CD25^−^) from the DN population, CD8 immature single positive (CD8ISP, CD3^−^ CD8^+^) and double positive (DP) stage (CD4^+^ CD8^+^). White symbols represent the sham group and grey symbols represent the bet group; circles or dots and squares were used for d1 and d4, respectively. Bars and symbols show the mean ± s.d. of n ≥ 5 mice (*p ≤ 0.05, **p < 0.01, Mann-Whitney test). (**C**) Percentage of viable CD4^+^ thymocytes, CD8^+^ thymocytes and γδ^+^ thymocytes. White symbols represent the sham group and grey symbols represent the bet group; circles or dots and squares were used for d1 and d4, respectively. Bars and symbols show the mean ± s.d. of n ≥ 5 mice (*p ≤ 0.05, **p < 0.01, Mann-Whitney test). (**D**) t-SNE representation of the cell abundance (density) for each thymic population analysed by flow cytometry at d1 and d4 after birth in control and bet groups. The plots show two representative animals per group.

To assess the *in vivo* effect of betamethasone in primary and secondary lymphoid organs, the weight of the thymus and spleen after birth, at day 1 (d1) and day 4 (d4), were determined. As expected, the weight of the thymus was lower in the betamethasone treated group when compared to the sham group (Δ_increase_ = −62.05%) at d1 (Fig. 4A). At d4, thymus weight was similar in both groups (Δ_increase_ = 0.76%), reflecting a recovery, as reported^14^. According to these data, the number of thymocytes obtained after mechanical disruption was significantly lower in the betamethasone group at d1 when compared to the sham group. At d4, the number of thymocytes was similar in both groups.

The effect of betamethasone in the cellular composition of the thymus after birth, at d1 and d4, was then analysed. Regarding thymocyte maturation, the percentages of thymocyte subsets were altered by prenatal betamethasone administration (Fig. 4B). As expected, double positive (DP) thymocytes, the most glucocorticoid-sensitive subset, were significantly reduced at d1 after treatment. This effect, however, was reversed at d4. A compensatory increase in the frequency of DN thymocytes was also observed at d1, but not at d4. Within the DN subpopulations, the first stages (DN1 and DN2) and the CD8 immature single positive (CD3^−^CD8^+^, CD8ISP) were the ones overrepresented in the betamethasone-treated samples at d1, while DN3 and DN4 percentages were maintained. At d4 after birth, the DN subpopulations were similar in treated and non-treated mice, except for the CD8ISP subset, which was significantly reduced in the betamethasone group.

Then, mature thymocytes subsets (single positive, SP) were examined (Fig. 4C). No effect of betamethasone was observed in the CD3^+^CD4^+^ T cell subset. As expected, the percentage of this subset increased at d4 when compared to d1, both in treated and control mice. In contrast, betamethasone hindered the increase in the percentage of CD3^+^CD8^+^ T cell subset observed at d4 in the control group. Finally, betamethasone increased the percentage of γδ^+^ T lymphocytes at d1, recovering normal values at d4. A t-SNE analysis, performed with the data obtained from flow cytometry, show the composition of the thymocyte compartment at d1 and d4 in treated and sham mice (Fig. 4D and Suppl. Fig. 4). The density plots confirm the above mentioned differences (Fig. 4B,C) in the betamethasone group when compared with the control group at both checkpoints.

**Figure 5:**
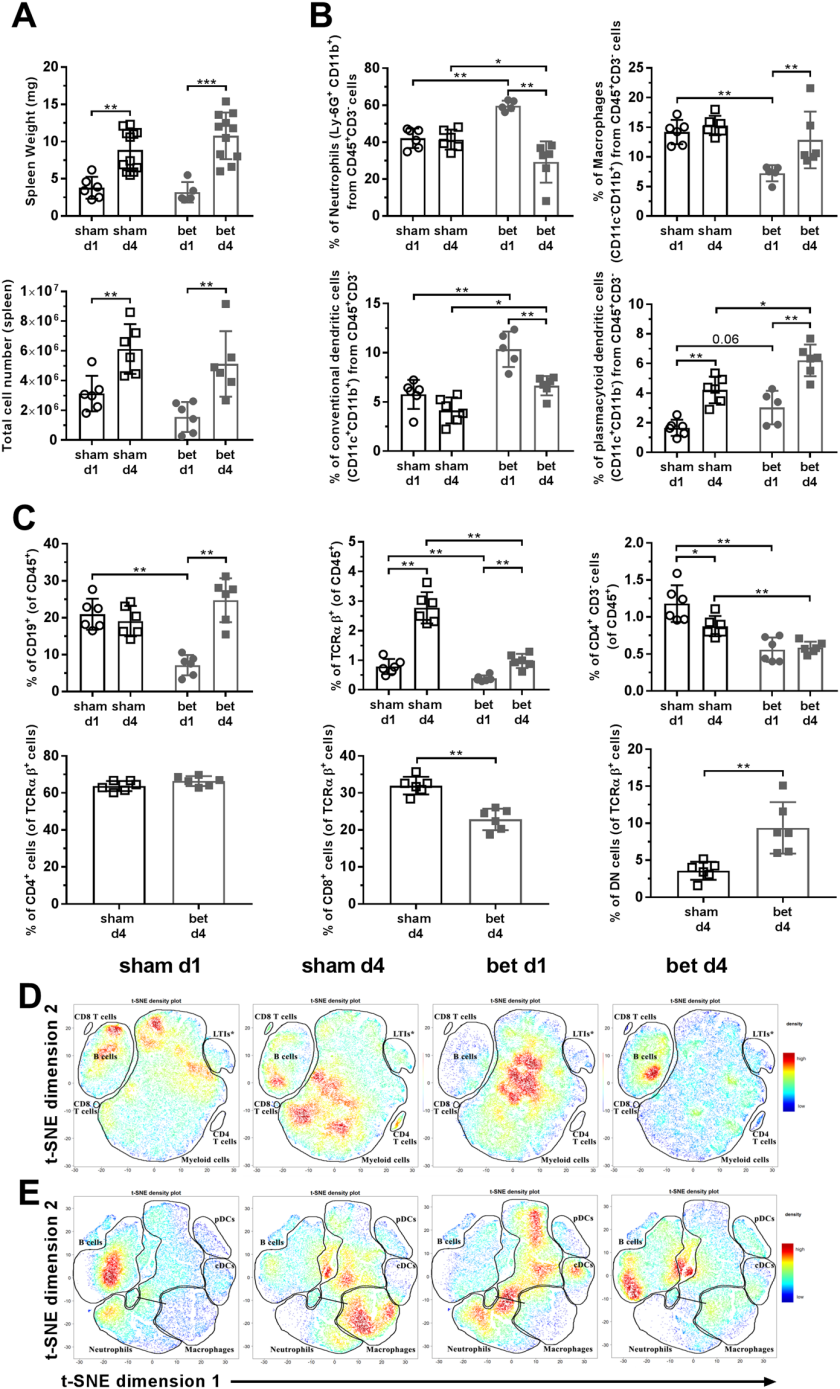
Immune cell subsets in the spleen are altered after betamethasone treatment. (**A**) Weight and total number of cells in the spleen. White symbols represent the PBS group and grey symbols represent the betamethasone (bet) group, circles or dots and squares were used for postnatal day 1 (d1) and postnatal day 4 (d4), respectively. Bars and symbols show the mean ± s.d. of n ≥ 5 mice (*p ≤ 0.05, **p < 0.01, ***p < 0.001, Mann-Whitney test). (**B**) Percentage of neutrophils (Ly6G^+^CD11b^+^), macrophages (CD11c^−^CD11b^+^), conventional dendritic cells (CD11c^+^CD11b^+^) and plasmacytoid dendritic cells (CD11c^+^CD11b^−^) from the CD45^+^ CD3^−^ population in the spleen. White symbols represent the sham group and grey symbols represent the bet group; circles or dots and squares were used for d1 and d4, respectively. Bars and symbols show the mean ± s.d. of n ≥ 5 mice (*p ≤ 0.05, **p < 0.01, Mann-Whitney test). (**C**) Percentage of viable lymphocytes (CD19^+^ B cells and TCRαβ^+^ T cells) and viable CD3^−^ CD4^+^. Percentages of CD4^+^ cells, CD8^+^ cells and DN cells from the T cell population are only available at d4. White symbols represent the sham group and grey symbols represent the bet group; circles or dots and squares were used for d1 and d4, respectively. Bars and symbols show the mean ± s.d. of n ≥ 5 mice (*p ≤ 0.05, **p < 0.01, Mann-Whitney test). (**D**) t-SNE representation of the cell abundance (density) for each spleen lymphoid population analysed by flow cytometry at d1 and d4 after birth in control and bet groups. Asterisk (*) refers to a population that is probably lymphoid tissue inducer cells (LTIs), but no positive marker in the cytometry panel was used. The plots show two representative animals per group. (**E**) t-SNE representation of the cell abundance (density) for each spleen myeloid population analysed by flow cytometry at d1 and d4 after birth in control and bet groups. The plots show two representative animals per group.

The weight of the spleen was not affected by betamethasone (Δ_increase_ = −16.32% at d1 and Δ_increase_ = 21.5% at d4) (Fig. 5A, upper panel). The cellular composition was also altered after prenatal treatment: neutrophils were markedly increased at d1 after betamethasone treatment (Fig. 5B) while the frequency of macrophages was clearly reduced. Conventional DCs showed higher frequencies at d1 and d4 after treatment, and and the frequencies of plasmacytoid DCs tended to the same increase.

Splenic lymphocyte subsets were then analysed (Fig. 5C). A decrease in the percentage of B lymphocytes (CD19^+^) was detected in the betamethasone group at d1 when compared to the sham group, but it subsequently normalised at d4. The TCRαβ^+^ T cell subset was reduced in percentage in the treated group at both time points. Likewise, the percentage of putative lymphoid tissue inducer cells (CD4^+^CD3^−^, LTIs) was reduced in the treated group at d1 and d4 when compared to the sham group. Due to the very low amount of T cells in spleen at d1, the T cell compartment was no further differentiated. At d4, no differences were found when comparing the CD4^+^ subset between the two groups, CD8^+^ cells were significantly decreased, and a compensatory increase in CD4^−^CD8^−^ in the betamethasone-treated group was observed. Similarly to the previous thymus analysis, a t-SNE analysis of the spleen was performed, including lymphoid (Fig. 5D and Suppl. Fig. 5) and myeloid (Fig. 5E and Suppl. Fig. 6) population density. A clear shift in the population density was observed in lymphoid and myeloid populations by the effect of betamethasone at both checkpoints.

In summary, betamethasone causes transient thymic hypotrophy, changes in the relative frequencies of splenic innate leukocytes and delayed seeding of T and B lymphocytes.

### Betamethasone induces a quiescent state in β-cells

**Figure 6:**
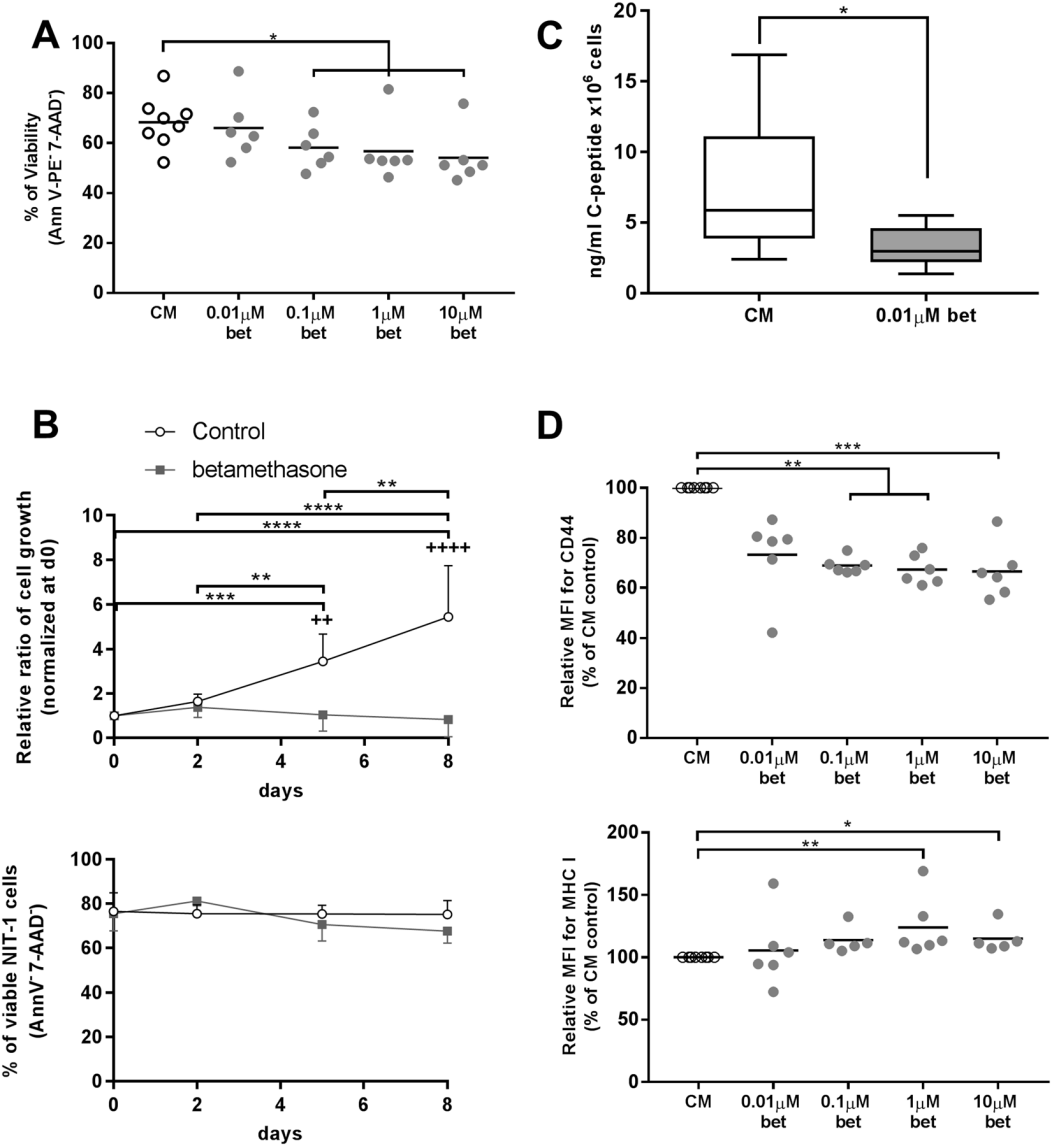
Betamethasone alters NIT-1 cells proliferation and C-peptide secretion. (**A**) Percentage of viable NIT-1 cells (annexinV PE^−^, 7aad^−^) after 48 h of culture with increasing betamethasone (bet) concentrations [0.01; 0.1; 1 and 10 μM] (grey dots). Basal medium conditions (white circles) were included. Lines show the mean of 6 independent experiments (*p ≤ 0.05, Dunn’s test, Kruskal-Wallis). (**B**) Upper panel: relative ratio of NIT-1 cell growth after 8 days of culture with 0.1 μM bet (grey squares) or without bet (white circles). Data presented as mean ± s.d. of relative cell number (n = 5 independent experiments), this being relative cell number of each day referred to their respective day 0 cell number (^+^p ≤ 0.05, ^++++^p < 0.0001 for betamethasone-control comparison; *p ≤ 0.05, ****p < 0.0001 for intragroup comparison, Tukey’s test, Two-way Anova). Lower panel: percentage of viable NIT-1 cell line (annexinV PE^−^, 7aad^−^) during the 8 days of culture. (**C**) C-peptide concentration in NIT-1 cells’ supernatant after 48 h without bet (white box) or with 0.01 μM bet (grey box). Data are represented as ng/ml × 10^6^cells/ml. Data shown as box and whisker plots (*p ≤ 0.05, Mann-Whitney test). (**D**) Relative median of fluorescence intensity (MFI) of CD44 and MHC class I in NIT-1 cells after 48 h of culture with bet at different concentrations [0.01; 0.1; 1 and 10 μM] (grey symbols) or without bet (white circles). Lines show the mean of n = 6 of relative MFI, this being MFI of each culture condition referred to their respective CM control (*p ≤ 0.05, **p < 0.01, ***p < 0.001 Dunn’s test).

To determine the effect of betamethasone in the target cells of T1D, a NOD-derived β-cell line, NIT-1, was exposed to increasing concentrations of this drug. After 48 h of culture, a dose-dependent reduction in the viability of NIT-1 was observed (Fig. 6A). To assess the effect of betamethasone in β-cell growth, NIT-1 cells were exposed to 0.1 μM betamethasone for 8 days (Fig. 6B, upper panel). NIT-1 cells doubled every 4 days in basal culture conditions but, in contrast, they completely lost their growth potential when exposed to betamethasone (Fig. 6B, upper panel). This effect was not due to a loss of viability (Fig. 6B, lower panel), but rather to cellular quiescence, since the effect was reversible after the removal of betamethasone (data not shown).

### Betamethasone impairs insulin secretion and induces phenotypical changes in β-cells

Insulin secretion was indirectly determined by measuring C-peptide concentration in culture media. In this sense, metabolic changes were observed in NIT-1 cells after 48 h culture with betamethasone at 0.01 μM. This concentration was chosen due to the high preservation of cell viability. Analysis of cell culture supernatants revealed a significant reduction in C-peptide concentration upon betamethasone treatment (Fig. 6C).

Because of their critical role in endogenous antigen presentation and autoimmunity in T1D^16,17^, cell surface expression of CD44 and MHC class I was determined in NIT-1 cells. CD44 expression was reduced when cells were exposed to betamethasone concentrations from 0.1 μM (Fig. 6D). In contrast, MHC class I was significantly overexpressed by the effect of betamethasone (1 μM) in comparison to control condition. In summary, betamethasone alters NIT-1 cell metabolism and phenotype.

### Betamethasone alters the transcription of genes related to autoimmunity, metabolism and islet mass in T1D target tissue

To determine putative changes in gene expression induced by betamethasone, 13 genes known to be related to T1D were selected: *Ins2*, *Gad1*, *Gad2* (autoantigens), *Cd44*, *Cd14*, *Ccl2*, *Il22ra1*, *Cxcl2* (related to autoimmunity), and *Tcf7l2*, *Pcsk1*, *Pten*, *Igfr1*, *Ptprj* (related to metabolism and islet cell mass).

**Figure 7:**
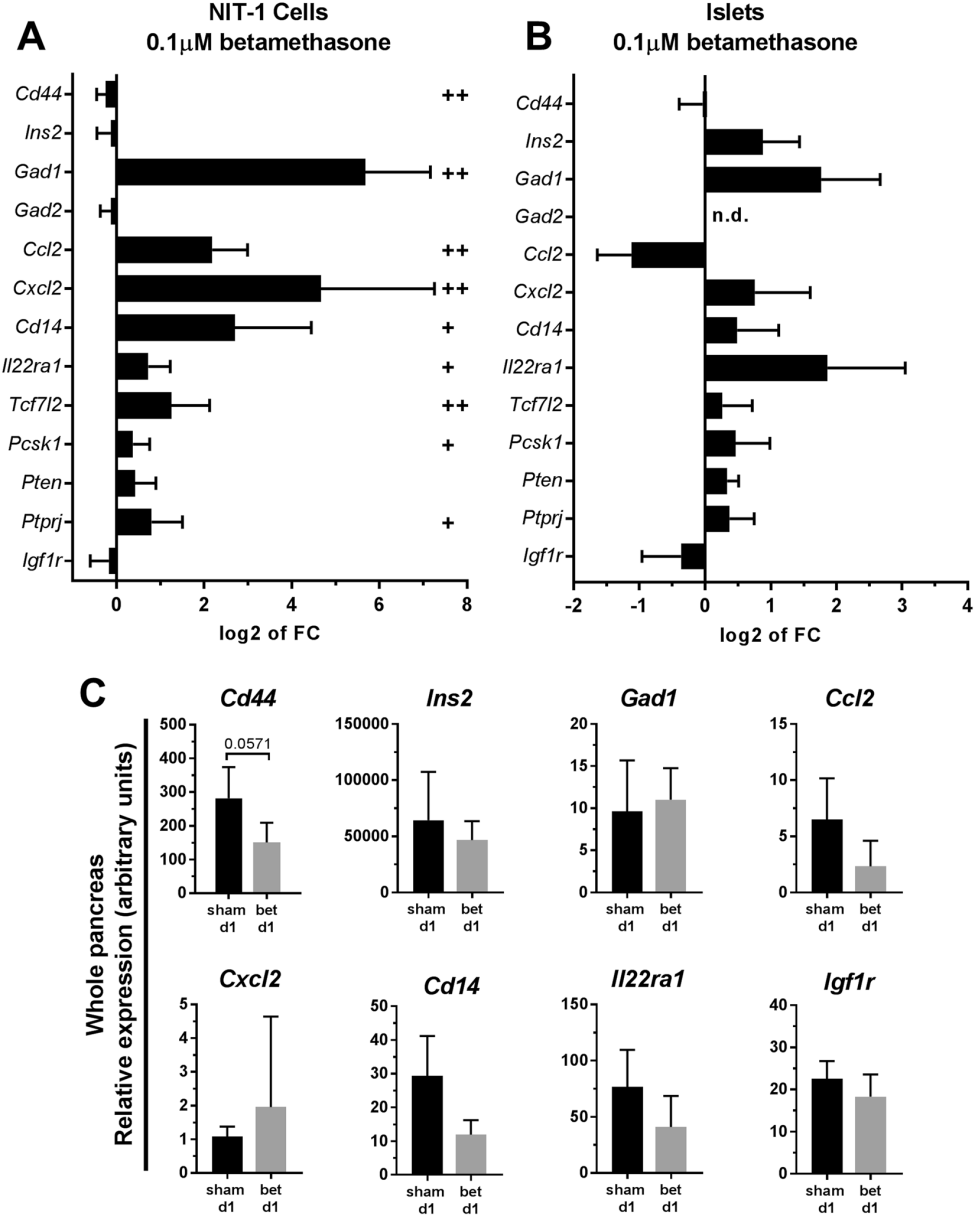
Betamethasone exposure alters gene expression in NIT-1 cells, islets of Langerhans and whole pancreas. (**A**) Relative gene expression of 13 selected genes in NIT-1 cells after betamethasone treatment (0.1 μM betamethasone, n ≥ 8) analysed by qRT-PCR. Gene expression was normalized to *Gapdh*. Bars show the mean ± s.d. of the Log2 of Fold Change (FC) using basal (complete medium-cultured NIT-1) transcription as standard value (^+^p ≤ 0.05 and ^++^p < 0.01, Wilcoxon test). (**B**) Relative gene expression of 13 selected genes in purified islets of Langerhans from 4 mice after culture with betamethasone (0.1 μM betamethasone, n = 4) analysed by qRT-PCR. Gene expression was normalized to *Gapdh*. Bars show the mean ± s.d. of the Log2 of FC using basal (complete medium-cultured islets) transcription as standard value (Wilcoxon test); n.d., not detected. (**C**) Histogram of relative gene expression of 8 selected genes in pancreases from newborn pups prenatally treated with 0.1 mg betamethasone (grey bars) or vehicle control (black bars) (n = 4 pancreases/group) analysed by qRT-PCR. Gene expression was normalized to *Gapdh*. Bars show the mean ± s.d. of gene expression (Mann-Whitney test).

After exposure to betamethasone, a significant upregulation of *Gad1*, *Ccl2*, *Cxcl2*, *Cd14*, *Il22ra1*, *Tcf7l2*, *Pcsk1*, and *Ptprj* and a significant downregulation of *Cd44* were observed in NIT-1 cells (Fig. 7A). No significant changes were found in the expression of *Ins2* and *Gad2*. As expected, a lower concentration of betamethasone (0.01 μM) caused fewer gene expression changes (Suppl. Fig. 7).

Given the differences between NIT-1 insulinoma cell line and islets in composition and function, we determined the effect of betamethasone in isolated islets from NOD mice (Fig. 7B). Betamethasone alters the transcription of the above mentioned genes. A similar gene expression profile was observed in treated islets when compared to NIT-1 cells. Remarkably, 10 out of 13 genes follow the same expression alteration that those observed in NIT-1 cells, and only *Ins2* and *Ccl2* were expressed in the opposite sense. These results, although non significant, show a biological effect of betamethasone in pancreatic islets.

Finally, the expression of eight selected genes after prenatal betamethasone administration was determined in pancreases from NOD pups, 1 day after birth. Figure 7C shows the biological effect, although non significant, of betamethasone in the whole pancreas. According to the effect of betametasone in the islets, *Cd44*, *Ccl2* and *Igfr1* genes were downregulated and *Gad1 and Cxcl2* were upregulated. By contrast, the expression of *Ins2*, *Cd14* and *Il22ra1* tended to diminish.

## Discussion

The incidence of T1D is steadily increasing, especially in children^2^. Changes in the environment are likely to contribute, but they are only starting to be identified^18^. A possible explanation is the environment during the perinatal development of the immune system. Prenatal administration of betamethasone is a common treatment used to improve survival of premature newborns. Glucocorticoids, however, are strong immune modulators^19^, but have short- and long-term effects on the immune system. Previous data have demonstrated that prenatal treatment with betamethasone induced changes in murine T cell TCR Vβ repertoire, altering the incidence of autoimmune diseases in the progeny^11^. In the case of T1D, protection from disease was observed in betamethasone-treated NOD offspring. The effect of betamethasone in experimental T1D has been expanded, not only in the immune system but also in the target cells of this disease, the insulin-producing β-cells. In the NOD mouse, betamethasone deteriorated splenocytes’ viability and caused a resistance to maturation in DCs, resulting in an inhibition of γδ^+^ T cell proliferation, suggesting a decrease in the immune response. Moreover, prenatal treatment with betamethasone treatment caused thymus and spleen hypotrophy and altered immune cell subsets in newborn NOD mice. Remarkably, betamethasone induced a quiescent state in β-cells, altered insulin secretion and caused phenotypical changes, thus affecting the immunogenicity of T1D target cells.

The thymic hypotrophy observed in newborn treated mice fits well with the *in vitro* toxic effect of betamethasone on thymocytes^20^. A delayed seeding of mature lymphocytes was observed in the spleen, in agreement with reported changes in haematopoiesis induced by glucocorticoid treatment, which favours myelopoiesis in detriment of lymphopoiesis^13^. Moreover, bone marrow-derived DCs, which are considered crucial antigen presenting cells involved in T1D, were unable to fully mature after *in vitro* betamethasone exposure, independently of the maturation stimuli, thus indicating that the treatment could impair their ability to promote an autoimmune reaction *in vivo*. This is the first demonstration of the effect of betamethasone in DCs in the context of T1D. The beneficial effect of glucocorticoids in autoimmune or autoinflammatory diseases has been previously described. For example, in atopic dermatitis, betamethasone acts on epidermal DCs, and inhibits Th2 response, thus ameliorating this inflammatory disease^21^. Moreover, it is well known that dexamethasone, a drug of the same family, induces a semi-mature phenotype in DCs^22–26^. Furthermore, DCs generated with betamethasone displayed a tolerogenic phenotype, based on the expression of molecules involved in antigen presentation, co-stimulation, and activation, as previously described^27^. These features fit well with the *in vivo* effect of betamethasone and are consistent with a decreased ability to stimulate autologous γδ^+^ T cell proliferation. Also, γδ^+^ T cells have been shown to possess a pathogenic role through IL-17 secretion^28^. The here reported results suggest that maturation-resistant DCs may arrest γδ^+^ T cell proliferation, which is crucial for the development of T1D. By abrogating the cross-talk between these two cell types^29^, T1D incidence can be reduced in NOD mice.

The abovementioned thymic and spleen hypotrophy induced by betamethasone correlates with altered subsets in these lymphoid organs. Alterations in leukocyte types were found after a birth-near injection of betamethasone in NOD pregnant females, consistent with the half-life of the drug. Some of this changes were transient but others were long-lived, as we recently described with long lasting changes in the TCR repertoire^11^. Moreover, normal outcome in thymic development is disrupted by betamethasone treatment^30^, altering DN and DP subsets by enhancing apoptosis of the highly sensitive DP compartment^31^ and blocking the cells in the CD8ISP stage thus further reducing the DP cells compartment in the betamethasone-treated offspring^32^, an effect also observed in this study. Interestingly, the detrimental effect of betamethasone in viability is only observed in the CD3^+^CD8^+^ thymocyte subset in the thymus and is shown in the periphery, fitting well with the cytotoxic role of this subset in autoimmunity against β-cells. Furthermore, the reduction in the LTIs subset in the spleen of treated mice would impair the establishment of tertiary lymphoid organs^33^, thus, in the pancreas, slowing insulitis and T1D. Innate immunity was altered by betamethasone, as demonstrated by the drastic increase in peripheral neutrophils, an effect reverted at d4. This effect, due to demargination of the neutrophils, was also reported on premature infants when betamethasone was administered shortly before delivery^34^. Demargination of neutrophils is caused by downregulation of adhesion molecules^35^ and changes in the actin matrix of the cell^36^. In summary, prenatal betamethasone effect in developing immune system might be responsible, at least in part, for leukocyte alterations in newborn mice.

The effect of betamethasone on target T1D cells was also evaluated in this study. Several studies suggest that β-cells have an active role in eliciting their own destruction^37,38^. Functional and phenotypical changes in insulin-producing cells are thought to be crucial in the loss of self-tolerance. Although being aware of the differences between primary β-cells and the NOD-derived NIT-1 cell line, it was not technically feasible to obtain islets of Langerhans from newborn pups. The results demonstrated that betamethasone deteriorated NIT-1 cell viability and arrested cell growth, thus preventing the exponential growth phase, as described in other cell lines^39,40^. This effect fits well with the decrease of C-peptide secretion caused by betamethasone, as indirect biomarker of insulin secretion, and with the described effect of synthetic glucocorticoids in islet β-cells, causing a reversible inhibition of insulin secretion^41^ but maintaining insulin synthesis. Transcriptomic experiments validate this result. Interestingly, betamethasone-stimulated MHC class I expression –similar to that observed in DC– may amplify endogenous antigen presentation but in combination with the decrease in CD44 expression in β-cells suggest a mechanism of immune evasion. CD44 was selected due to its function in the regulation of cell adhesion and migration, both in insulitis^16^ and in the development of experimental disease^17^. Moreover, anti-CD44 treatment improved insulin production in islet transplantation^42^ by reducing graft-infiltrating T cells. Since the expression of CD44 in β-cells is increased in the pancreases of T1D patients, mainly at T1D onset^43^, thus increasing the susceptibility to autoimmune destruction^44^, the betamethasone-induced downregulation of *Cd44* gene observed in this study may contribute to β-cells protection in the context of autoimmunity.

The phenotypic and functional effects of betamethasone was accompanied by changes in the expression of genes related to autoimmunity, metabolism and islet mass, both in NIT-1 cells and islets such as a downregulation of *Igf1r* and a hyperexpression of *Ptprj* –both genes related to insulin sensing^45^. Moreover, overexpression of the tumour suppressor gene *Pten* explains the inhibition of the *in vitro* cell growth caused by betamethasone^46^. The increased expression of *Cd14* gene, a pattern recognition receptor, suggests a homeostatic mechanism induced by cellular-stress to better regulate insulin secretion^47^. However, no further effect was found in NIT-1 cells due to the absence of LPS, a ligand of the CD14 protein, in the cell culture. Betamethasone may also affect β-cell mass homeostasis by upregulating *Il22ra1* gene, which induces the expression of Regenerating (*Reg*) genes through the effect of IL-22^48^. Again, the absence of the ligand in culture conditions could prevent further expansion.

Betamethasone also altered the expression of *Ccl2* gene in T1D target tissue. This chemokine is involved in the recruitment of monocytes, macrophages, DCs, and activated T-cells, thus leading to islet destruction^49^ or tolerance^50^. *Ccl2* gene was downmodulated in betamethasone-treated islets and in the pancreas of prenatally-treated pups, correlating with the reduction of destructive insulitis. The increased *Ccl2* gene expression observed in NIT-1 cells can be due to its tumoral origin. Another important chemokine in T1D is Cxcl2. *Cxcl2* gene was overexpressed by betamethasone in the three different substrates, indicating an increase of neutrophil recruitment to T1D target tissue. Neutrophils have an important role in β-cell destruction when accompanied by T cells in the microenvironment^51^. However, the here reported data suggest that T cell recruitment is halted by *Ccl2* downmodulation. In this scenario, and because apoptotic islet cells removal by neutrophils has anti-inflammatory effects, neutrophils would contribute to reduce inflammation^52^. This alteration fits well with the increased percentages of splenic DCs and neutrophils after betamethasone treatment.

Furthermore, *Gad1* gene –codifying for GAD67 autoantigen^53^– was strongly upregulated by betamethasone. It has been reported that glucocorticoids upregulate GAD67^54^. This is the most expressed isoform of GAD. The other isoform, GAD65 is a target autoantigen of very early autoimmune attack in NOD mice^55^ that is not affected by betamethasone. The role of GAD67 is less clear but immunization of prediabetic NOD mice with GAD67 prevents the onset of diabetes^56^. The here reported hyperexpression of *Gad1*, toghether with thymocytic apoptosis, suggests immunomodulatory effects.

It has been previously demonstrated that prenatal administration of betamethasone causes changes in the TCR repertoire influencing the outcome of autoimmunity. The results from this study confirm that other relevant cell types in T1D are affected. Since NOD strain is diabetes-prone, the alteration of the immune system cells disrupts this predisposition. Moreover, the additional effect of betamethasone in reducing β-cell immunogenicity may explain the prevention of the disease by this drug.

Since autoimmune diabetes is different in NOD mice and in humans, it would be interesting to analyse the effects of prenatal administration of betamethasone in children: whether it has the same protective effect or it predisposes to autoimmunity. In this sense, a study performed in a Danish cohort of children studied the effects of the prescription of several corticosteroids to pregnant women^57^ according to the route of administration (systemic, inhaled or topical). The results showed a weak increase of the risk of developing T1D in the offspring, but the effects of each corticosteroid was not individually analyzed in this study. We found no specific studies that addressed the effect of the administration prenatal betamethasone in the development of T1D. Further studies should be performed in children prenatally exposed to betamethasone between the week 24 and 34 of pregnancy^58^ to determine the prevalence of autoimmune diseases.

Taking into account that the immune system of the newborn is developing^59^, the prenatal immunological remodelling makes the immune system very susceptible to drugs. In fact, it has been reported that prenatal treatment with betamethasone has immunomodulatory effects in newborn infants in terms of acceleration of NK activity, decrease of T cell proliferation, and inappropriate cellular immune responses^60^. These effects last for weeks, and may be harmful for the newborn. Concerns have been raised about the effects of prenatal betamethasone exposure on the developing immune system. In this sense, the amount of betamethasone administered to pregnant women (2 doses of 12 mg) corresponds approximately to 900 nM (for 70 Kg of body weight), and this concentration has no *in vitro* deleterious effect in human leukocyte viability, suggesting that betamethasone is not detrimental for human leukocytes in prenatal treatment.

Given the increase in the incidence of allergies and autoimmune disorders in the last years, elucidating the mechanisms of action of prenatal glucocorticoids will allow the design of prevention strategies. In this sense, the results of this study show that the effect of betamethasone on the developing immune system influences the susceptibility to experimental T1D, by direct effect to immune system cells and to pancreatic β-cells. Understanding the effect of betamethasone in self-tolerance could have potential clinical relevance in T1D.

## Material and Methods

### Mice breeding and treatment

Wild-type NOD mice, genetically prone to spontaneously develop autoimmune T1D, were obtained from The Jackson Lab (Bar Harbor, ME, USA) and kept under specific pathogen-free conditions. T1D incidence in NOD females was 67%. Mice were mated and the presence of a vaginal plug was considered day 0.5 of pregnancy (E0.5). On day 20 (E20) mice were i.p. injected with 0.1 mg betamethasone (Sigma-Aldrich, St. Louis, MO, USA) in Phosphate Buffered Saline (PBS, Oxoid Limited, Hampshire, UK) or vehicle control (PBS, Oxoid Limited, plus 10% EtOH, Alcoholes Gual, Pacs del Penedès, Spain). Betamethasone (Sigma-Aldrich) was diluted in ethanol (EtOH, Alcoholes Gual) at 5 mg/mL. For further dilution, PBS (Oxoid Limited) was used.

### Pancreases, islets and cell line

Newborn pups prenatally treated with betamethasone and controls (4 mice/group) were euthanised at days 1 and 4. Pancreas was removed at day 1 (48 h after betamethasone administration) and snap frozen in an isopentane/cold acetone bath for RNA isolation.

To isolate islets, pancreases from NOD mice were perfused with collagenase (Collagenase P, Roche, Indianapolis, IN) through the common bile duct^61^. Pancreases were removed and incubated at 37 °C for 30 min. After digestion, islets were hand-picked and groups of 10–20 islets were cultured. Culture medium (CM) was RPMI-1640 (Biowest, Nuaille, France) with 10% Foetal Bovine Serum (Gibco, Invitrogen, Carlsbad, CA, USA), 100 U/mL penicillin (Normon SA, Madrid, Spain), 100 μg/mL streptomycin (Reig Jofre, Sant Joan Despí, Spain), 2 mmol/L glutamine (Sigma-Aldrich), 1 mmol/L sodium pyruvate (Gibco), and 25 μmol/L β-mercaptoethanol (Sigma-Aldrich).

NIT-1 cell line, established from NOD/Lt mice^62^, was chosen because of its similarity to β-cells in terms of autoantigens (American Type Culture Collection, Manassas, VA, USA) and cultured in CM. Viability was assessed by staining with annexinV (AnnV) PE (Immunotools, Fryesoythe, Germany) and 7-amino-actinomycin D (7aad) labelling (BD Pharmingen, San Diego, CA, USA) and analysed with FACS Canto II (BD Biosciences, San Jose, CA, USA).

### Flow cytometry analysis of cells from newborn mice lymphoid organs

To assess the changes induced by prenatal betamethasone in leukocyte subsets of newborn mice, thymi and spleens of pups euthanised at d1 and d4 were harvested and weighed. Single-cell suspensions were obtained by mechanical disruption. Cells were stained for flow cytometry (FACS Canto II, BD Biosciences) analysis with anti-CD3e eFluor 450 (eBioscience, San Diego, CA, USA), anti-CD4 APC (BD Biosciences), anti-CD4 APC-Cy7 (eBioscience), anti-CD8α PE-Cy7 (eBioscience), anti-CD11b APC (eBioscience), anti-CD11c PE-Cy7 (BD Biosciences), anti-CD19 V450 (BD Biosciences), anti-CD25 PE (BioLegend, San Diego, CA, USA), anti-CD44 APC (eBioscience), anti-CD45 APC-Cy7 (eBioscience), anti-TCRγδ chain PerCP-Cy5.5 (BioLegend), anti-TCRαβ chain PerCP-Cy5.5 (BioLegend), anti-Ly6g PerCP-Cy5.5 (BD Biosciences) and Pacific Orange dye (ThermoFisher Scientific, Waltham, MA, USA) for viability. Data were manually analysed using FlowJo software (Tree Star Inc., Ashland, OR, USA). The dimensionality reduction algorithm t-SNE (t-distributed stochastic neighbour embedding) in R, using the CRAN package “Rtsne”, was used for direct comparison of treated and untreated samples.

### *In vitro* toxicity assay

To study the effect of betamethasone, spleens from prediabetic NOD mice (8–10 weeks of age) were harvested and single-cell suspensions were obtained by mechanical disruption. After haemolysis, cell number and viability were determined by AnnV PE (Immunotools), 7aad (BD Biosciences) and Perfect Count Microspheres (Cytognos SL., Salamanca, Spain) using flow cytometry (FACS Canto II, BD Biosciences). Splenocytes were cultured at 10^6^ cells/mL with 0.1 nM, 1 nM, 2.5 nM, 5 nM, 10 nM, 50 nM, 100 nM or 1000 nM betamethasone or the corresponding controls (PBS). Cell viability was determined as abovementioned at 24 h using flow cytometry. Human PBMCs were obtained from 10 mL blood sample by means of Ficoll Paque (GE Healthcare, Marlborough, MA, USA) density gradient centrifugation. PBMCs were cultered at 10^6^ cells/mL with 0.1 nM to 10^6^ nM betamethasone. After 48 h, cell viability was determined in PBMCs using flow cytometry as detailed above, and three leukocyte subsets were analyzed using anti-CD3 APC, anti-CD14 APC-Cy7 and anti-CD19 PE (Immunotools).

### Effect of betamethasone on DCs phenotype

DCs were derived from bone marrow precursors after 7 days of culture with mGM-CSF (Prospecbio, Rehovot, Israel), with or without betamethasone (Sigma-Aldrich) from day 3 until the end of the differentiation protocol. Bone marrow was obtained from femur and tibia bones. After haemolysis, cell number and viability were determined by AnnV PE (Immunotools), 7aad (BD Biosciences) and Perfect Count Microspheres (Cytognos SL) using flow cytometry. Wells were seeded with 10^6^ cells/mL in CM with 1000 IU/mL mGM-CSF, and 50% of culture media was replaced on days 3, 5, 7 and 8 with CM supplemented with 10% bovine foetal serum (Gibco), 2 mmol/L glutamine (Sigma-Aldrich), 1 mmol/L sodic pyruvate (Gibco) and 2000 UI/mL mGM-CSF (Prospecbio). Betamethasone was added at increasing concentrations [10 nM, 100 nM and 1000 nM] at days 3–7. On day 8, DCs were cultured with insulin (Sigma-Aldrich) at 20 µg/mL for 2 h and then matured with 100 ng/mL LPS (Sigma-Aldrich) for 24 h or 1 µg/mL CpG (Invivogen, San Diego, CA, USA) for 48 h. Untreated DCs were considered iDCs.

To determine the phenotype, iDCs, mDCs, and mDCs generated with betamethasone at increasing concentrations (10betDCs, 100betDCs, and 1000betDCs) were harvested with accutase (eBioscience), stained with anti-CD11c PE-Cy7 (BD Biosciences), anti-CD25 PE (eBioscience), anti-CD40 APC (BD Biosciences), anti-CD86 PE (eBioscience), anti-Class I MHC eFluor 450 (eBioscience), anti-Class II MHC APC (eBioscience), AnnV PE (Immuntools), and 7aad (BD Biosciences), and analysed by flow cytometry (FACS Canto II, BD Biosciences) using FlowJo software (Tree Star Inc.). Corresponding fluorescence minus one staining was used as control.

### Proliferation assays

To determine the effect of betamethasone on lymphocyte proliferation, splenocytes from NOD mice were stained with 0.31 µM CFSE (ThermoFisher Scientific) and 2 × 10^5^ splenocytes were seeded and cultured with 1 nM, 5 nM, 25 nM betamethasone. To assess the proliferation capacity of splenocytes, 25 ng/mL PMA (Sigma-Aldrich) and 250 ng/mL Ionomycin (IO, Sigma-Aldrich) were added. After 3 days, the cells were harvested and stained with anti-CD3 V450 (BD Bioscience), anti-CD19 PE (BD Bioscience) and 7aad (BD Bioscience) to assess viability and lymphocyte subsets by flow cytometry (LSR Fortessa, BD Biosciences).

To determine the tolerogenic capacities of DCs generated under the effect of betamethasone, CFSE-stained splenocytes were co-cultured with autologous iDCs, mDCs, and with mDCs differentiated in the presence of increasing concentrations of betamethasone at a 10:1 ratio (10^5^ splenocytes:10^4^ DCs) for 4 days; CM and 25 ng/mL PMA plus 250 ng/mL IO were used as negative and positive controls, respectively. Cells were stained with anti-CD3 V450 (BD Biosciences), anti-CD4 APC-Cy7 (BD Biosciences), anti-CD8 V500 (BD Biosciences), anti-CD19 PE (BD Biosciences), anti-TCRγδ PE-Cy7 (BioLegend), and 7aad (BD Biosciences) to assess the viability and the different lymphocyte subsets by flow cytometry (LSR Fortessa, BD Biosciences). Culture supernatant was collected and frozen at −80 °C until use. The concentration of IL17A after the co-culture of splenocytes with DCs was assessed using a Ready-Set-Go IL17A ELISA kit (Invitrogen).

### *In vitro* effect of betamethasone on β-cells

To further explore betamethasone effect, NIT-1 and isolated islets cells were cultured with CM with or without betamethasone (100 nM, Sigma-Aldrich) for 2–10 days. CM was replaced every 2-3 days.

At least 5 × 10^5^ NIT-1 cells were cultured for 2 days with graded concentrations of betamethasone (up to 10 μM) or CM. Cells were harvested using 0.05% trypsin EDTA (ThermoFisher) and stained for anti-CD44 BV786 (BD Biosciences), anti-Class I MHC eFluor 450 (eBioscience), AnnV PE (Immunotools) and 7aad (BD Biosciences). Median Fluorescence Intensity (MFI) and viability were assessed using flow cytometry (LSR Fortessa, BD Biosciences). Corresponding fluorescence minus one staining was used as control. Data were analysed using FlowJo software (Tree Star Inc.). Culture supernatant from NIT-1 cells was frozen at −80 °C until use. Insulin secretion was assessed by measuring C-peptide concentration in the supernatant by ELISA (RayBiotech, Norcross, GA, USA).

### Quantitative RT-PCR

To determine the effects of betamethasone on transcriptome, quantitative RT-PCR was performed. Briefly, RNA was isolated from NIT-1 cells, purified islets and whole pancreases using RNeasy Micro Kit (QIAGEN, Hilden, Germany), and was reverse-transcribed with a High Capacity cDNA Reverse Transcription Kit (ThermoFisher Scientific). The cDNA synthesis reactions were carried out using random hexamers (0.5 mg/mL, BioTools, Madrid, Spain) and reverse transcriptase Moloney-murine-Leukaemia-virus (200 U/mL, Promega, Madison, WI, USA). For purified islets, targeted cDNA was pre-amplified prior to quantitative real-time PCR with TaqMan PreAmp Master Mix (ThermoFisher Scientific). Quantitative RT-PCR assays were performed by TaqMan universal assay (ThermoFisher Scientific) on a LightCycler® 480 (Roche, Mannheim, Germany) using the following TaqMan assays: *Ins2* (Mm00731595_gH), *Gad1* (Mm04207432_g1), *Gad2* (Mm00484623_m1), *Cd44* (Mm01277161_m1), *Ccl2* (Mm00441242_m1), *Cxcl2* (Mm00436450_m1), *Ptprj* (Mm00501277_m1), *Pten* (Mm00477208_m1), *Igf1r* (Mm00802831_m1), *Cd14* (Mm00438094_g1), *Pcsk1* (Mm00479023_m1), *Tcf7l2* (Mm00501505_m1), *Il22ra1* (Mm01192943_m1). Relative quantification was performed by normalising the expression for each gene to that of the housekeeping gene *GAPDH* (Mm99999915_g1), as described in the 2^−ΔCt^ method^63^. Values from NIT-1 and islets were normalised using their respective CM controls. The genes were selected using a gene-interaction database (Harmonizone)^64^ of glucocorticoid receptor, the list of genes differentially expressed in T1D patients^43,65^ and other β-cell specific genes^66^.

### Statistical Analysis

Statistical analysis was performed using Prism 7.0 software (GraphPad software Inc., San Diego, CA, USA). Analysis of variance (Friedman’s test) was used for comparisons with several factors using a post-hoc Dunn’s test for the comparison between groups. For unpaired data, a non-parametric Mann-Whitney test was used; for paired data, non-parametric Wilcoxon test was used. A sigmoidal non-linear regression was used for IC50 assessment.

### Ethics Statement

This study was carried out in strict accordance with the recommendations in the Guide for the Care and Use of Laboratory Animals of the Generalitat de Catalunya, Catalan Government. The protocol was approved by the Committee on the Ethics of Animal Experiments of the Germans Trias i Pujol Research Institute (Permit DAAM 8948) and has followed the principles outlined in the Declaration of Helsinki for animal experimental investigation. For human samples, all subjects gave written informed consent in accordance with the Decaration of Helsinki and the study was carried out after the approval and in strict accordance with the guidelines of Germans Trias i Pujol Research Institute and Hospital Ethical Committee.

## Supplementary information

Supplementary Figures

## Data Availability

The datasets generated and/or analysed during the current study are available from the corresponding author on reasonable request.
