# Supplementary material for: Prenatal Betamethasone interferes with immune system development and alters target cells in autoimmune diabetes
Source: Sci Rep. 2019 Feb 4;9:1235. doi: 10.1038/s41598-018-37878-9 (PMC6362293; doi:10.1038/s41598-018-37878-9)
Supplement: Supplementary file 1 — Supplementary Figures [file 41598_2018_37878_MOESM1_ESM.pdf]

SREP-18-19439B

Prenatal Betamethasone interferes with immune system development and alters target cells in autoimmune diabetes

David Perna-Barrull[1], Silvia Rodriguez-Fernandez[1], Irma Pujol-Autonell[1], Anna Gieras[2], Rosa M Ampudia-Carrasco[1], Adrian Villalba[1], Laura Glau[2], Eva Tolosa[2], Marta Vives-Pi[*1,3]

[1]Immunology Section, Germans Trias i Pujol Research Institute, Autonomous University of Barcelona, Badalona, Spain, [2]Department of Immunology, University Medical Center Hamburg-Eppendorf, Hamburg, Germany, [3]CIBERDEM, Barcelona, Spain

[*]**Author for correspondence**: mvives@igtp.cat

Supplementary Figure 1

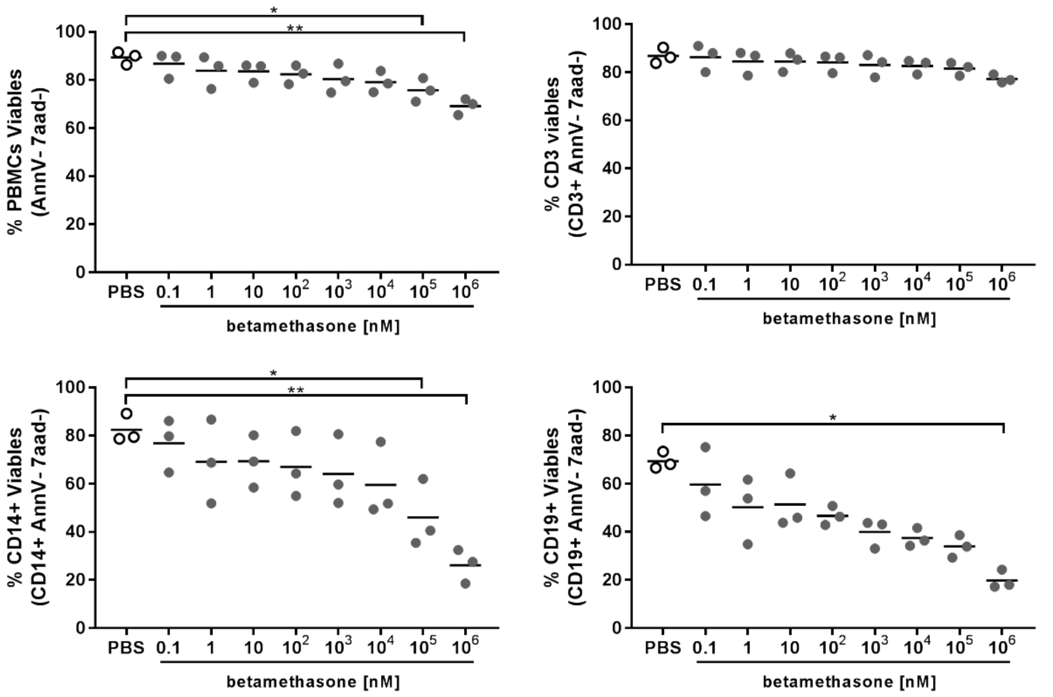

Supplementary Figure 1. Betamethasone impairs human leukocytes viability at high concentration *in vitro*. Percentage of viable human peripheral blood leukocytes (annexinV PE^-, $7aad^-$) after 48 h of culture with betamethasone [0.1 nM to 10^6 nM], and control condition (PBS). Lines show the mean of 3 independent experiments ($*p \leq 0.05$ and $**p < 0.01$, Dunn's test, Kruskal-Wallis).

Supplementary Figure 2

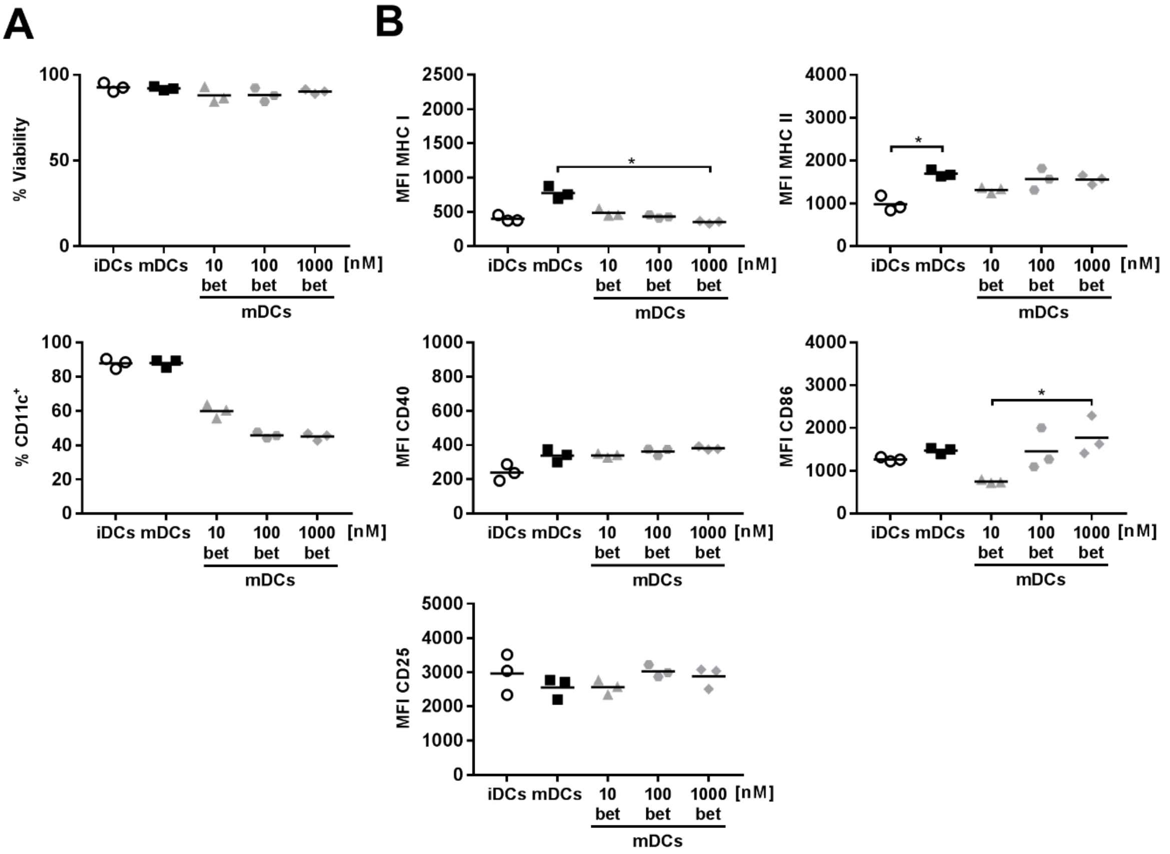

Supplementary Figure 2. Dendritic cells (DCs) derived from bone marrow precursor cells in the presence of betamethasone show a semi-mature phenotype after CpG stimuli. (**A**) Upper panel: Percentage of viability of DCs (annexinV PE^{-}, $7aad^{-}$ of $CD11c^{hi}$). Lower panel: Differentiation yield of DCs from bone marrow progenitors (% $CD11c^{+}$). (**B**) Median of fluorescence intensity (MFI) of MHC class I, MHC class II, CD40, CD86 and CD25 surface expression on DCs ($CD11c^{+}$). White circles represent immature DCs (iDCs) after differentiation. Black and grey symbols represent DCs stimulated with CpG (1 µg/mL) for 48 h, without betamethasone (bet) (mDCs, black squares), or with 10 nM bet (grey triangles), 100 nM bet (grey dots), 1000 nM bet (grey rhombus). Lines show the mean of 3 independent experiments (*$p \leq 0.05$, Dunn's test, Friedman test).

Supplementary Figure 3

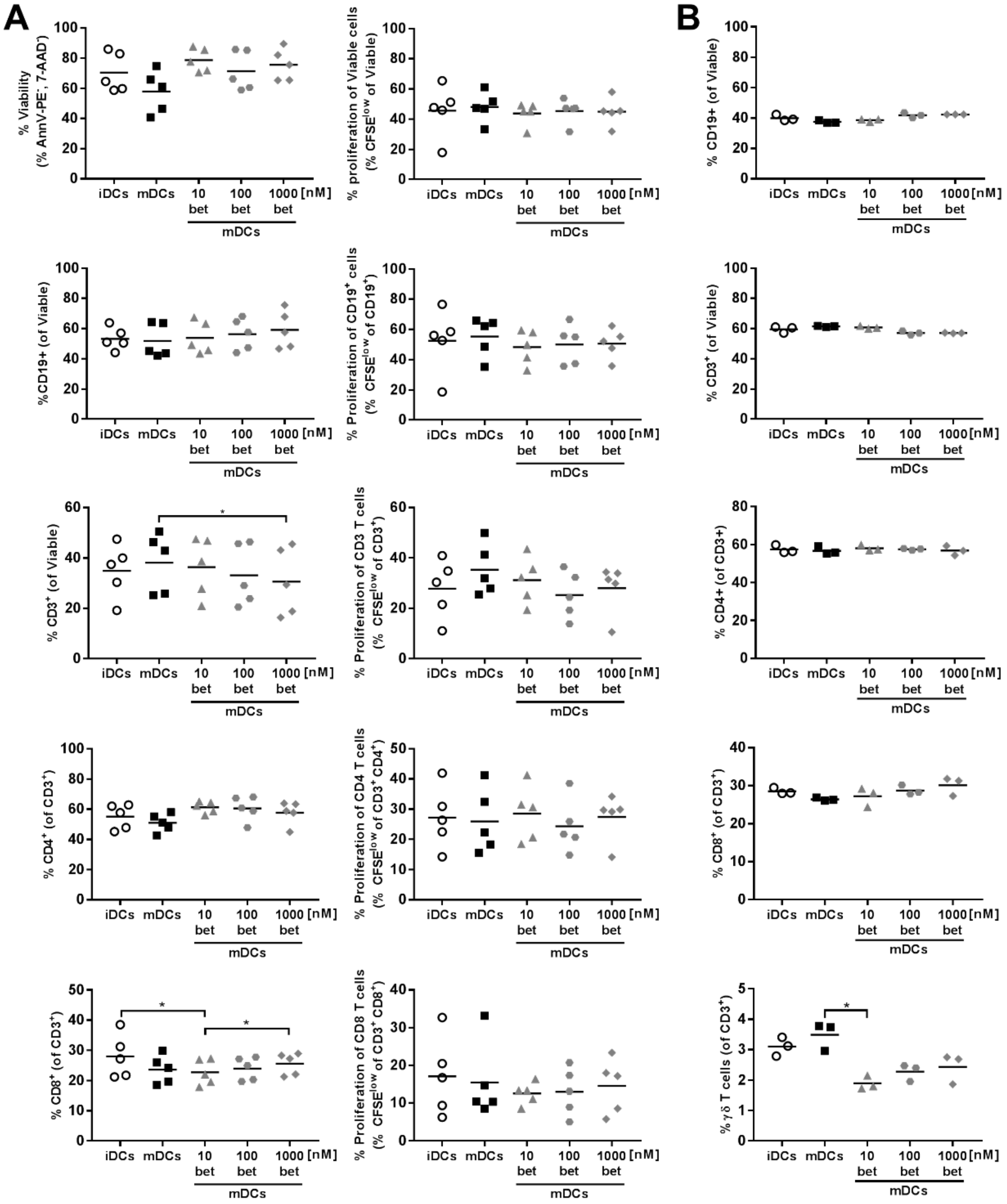

Supplementary Figure 3. B and T cell viability and proliferation is not altered in co-cultures with DCs treated with betamethasone. DCs were loaded with insulin (20 μg/ml), after LPS or CpG stimulation, and were cultured with splenocytes (1:10) for 4 days. White circles show immature DCs (iDCs), black squares show mature DCs (mDCs) generated in basal conditions and stimulated with LPS or CpG, grey symbols show mDCs with 10 nM (triangles), 100 nM (dots) and 1000 nM (rhombus) betamethasone during differentiation. (**A**) Left column: percentage of viable cells, of $CD3^+$ T cells and $CD19^+$ B cells within viable population and $CD4^+$ and $CD8^+$ T cells within $CD3^+$ population after co-culture with DCs stimulated with LPS. Right column: percentage of autologous proliferation of viable cells, of $CD3^+$ T cells and $CD19^+$ B cells within viable population and $CD4^+$ and $CD8^+$ T cells within $CD3^+$ population (%

CFSE^{Low}) after co-culture with DCs stimulated with LPS. Lines show the mean of 5 independent experiments (*$p \leq 0.05$, Dunn's test, One-Way ANOVA). (**B**) Percentage of $CD3^+$ T cells and $CD19^+$ B cells within viable population and $CD4^+$, $CD8^+$ and $\gamma\delta^+$ T cells within $CD3^+$ population after co-culture with DCs stimulated with CpG (representative experiment) (*$p \leq 0.05$, Dunn's test, Kruskal-Wallis).

Supplementary Figure 4

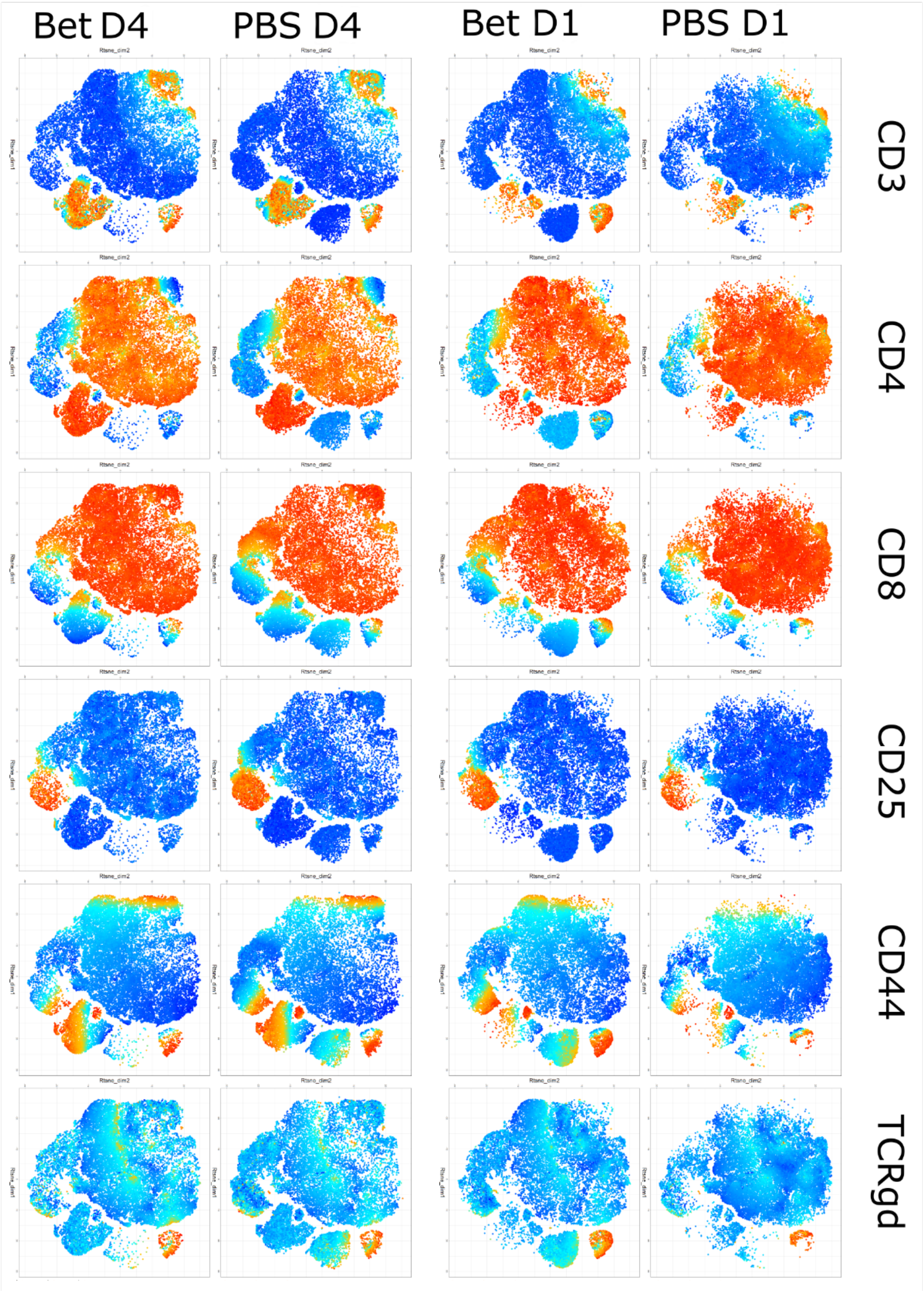

Supplementary figure 4. Alterations in thymic subsets after betamethasone treatment. T-SNE representation of the surviving cells in the thymic compartment after prenatal betamethasone treatment according to the expression of CD3, CD4, CD8, CD25, CD44 and TCRγδ. Each dot represents a cell and the colors show levels of expression of the indicated markers. The plots show two representative animals per group.

Supplementary Figure 5

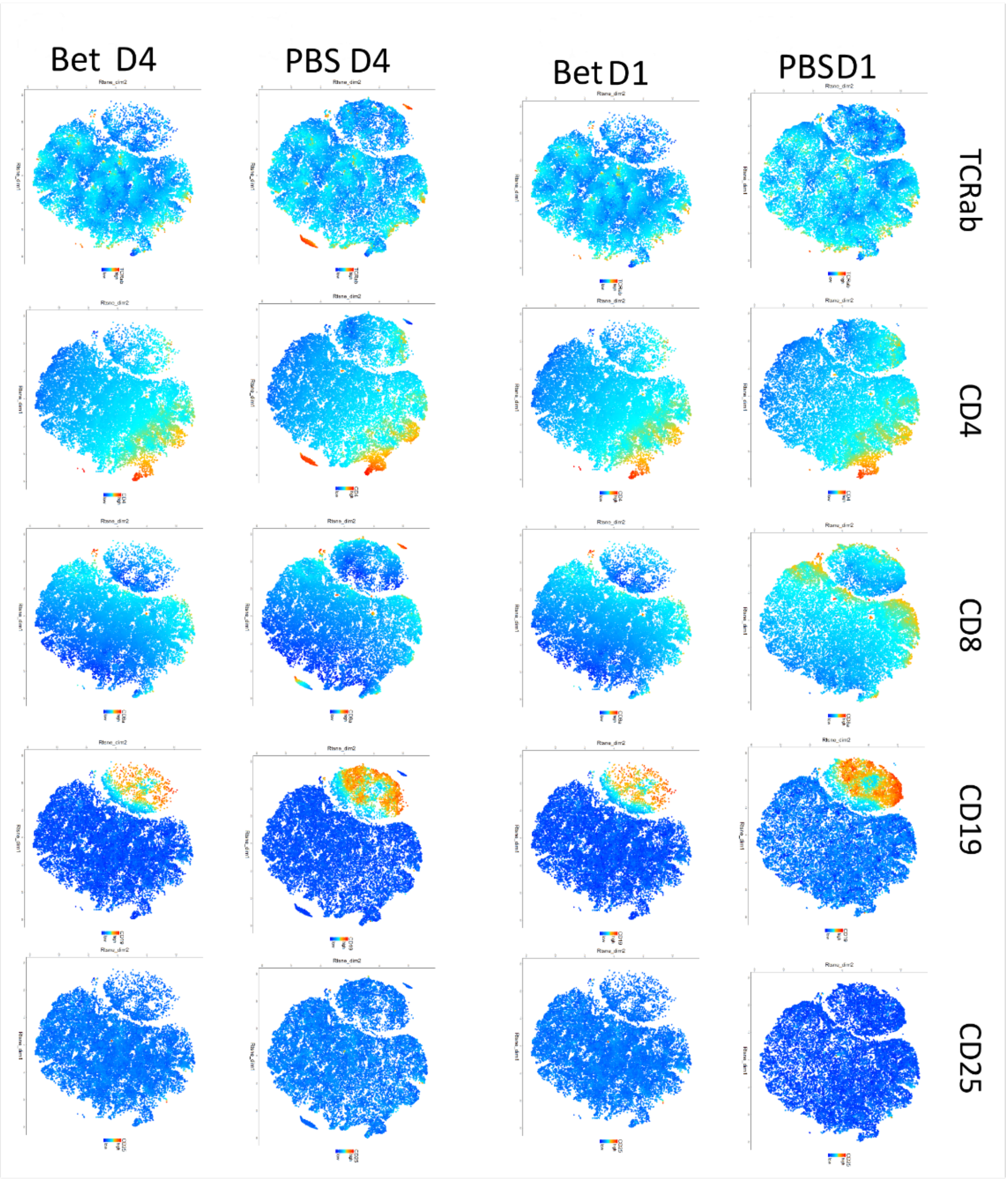

Supplementary figure 5. Alterations in spleen lymphoid subsets after betamethasone treatment. T-SNE representation of the surviving cells in the spleen lymphoid compartment after prenatal betamethasone treatment according to the expression of TCRαβ, CD4, CD8, CD19 and CD25. Each dot represents a cell and the colors show levels of expression of the indicated markers. The plots show two representative animals per group.

Supplementary Figure 6

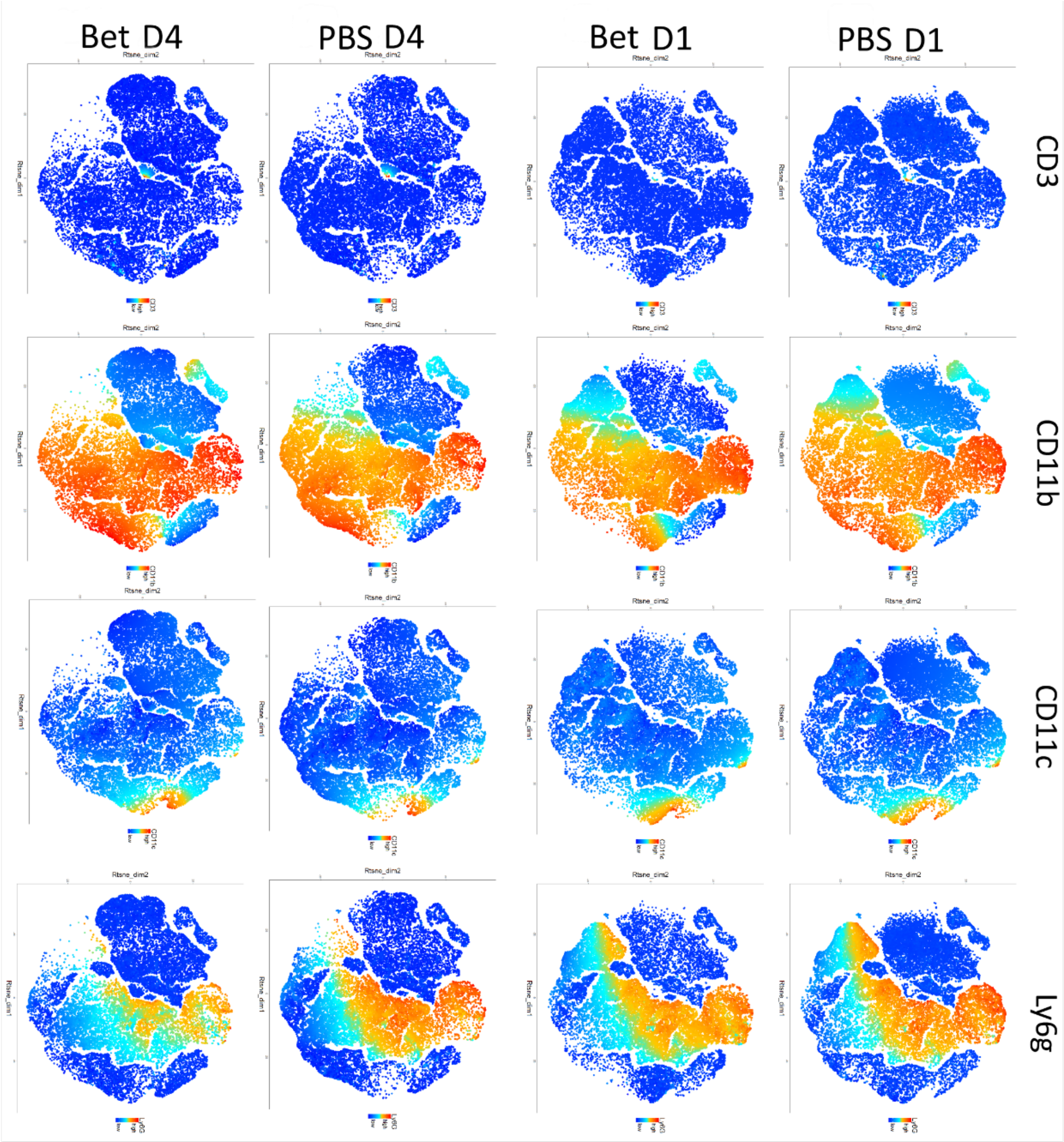

Supplementary figure 6. Alterations in spleen myeloid subsets after betamethasone treatment. T-SNE representation of the surviving cells in the spleen myeloid compartment after prenatal betamethasone treatment according to the expression of CD3, CD11b, CD11c and Ly6g. Each dot represents a cell and the colors show levels of expression of the indicated markers. The plots show two representative animals per group.

Supplementary Figure 7

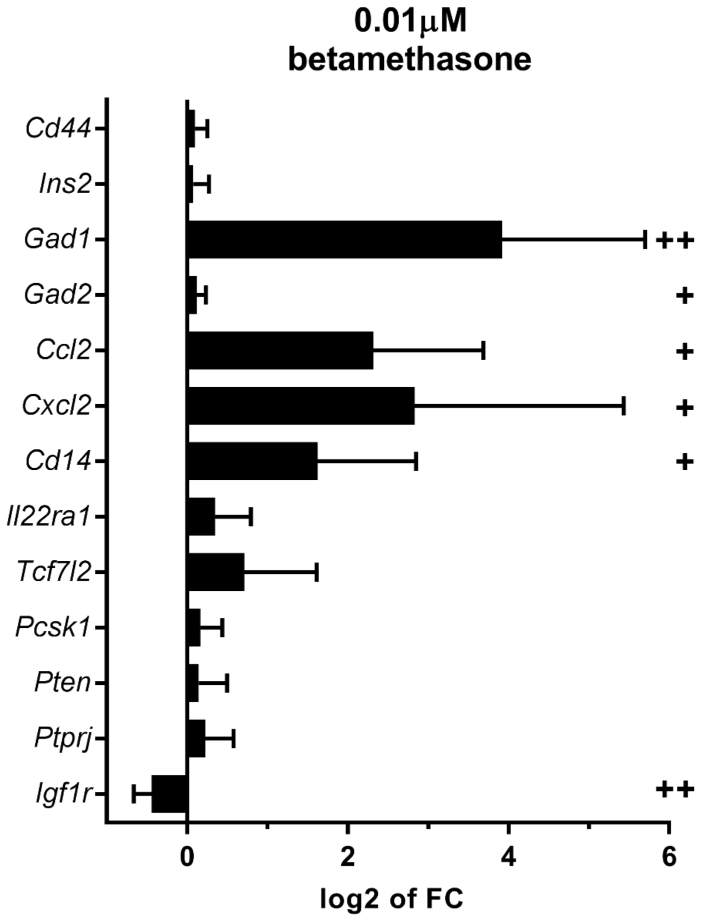

Supplementary Figure 7. Gene expression changes induced by 0.01 μM betamethasone concentration in NIT-1 cells**.** Relative gene expression of 13 selected genes in NIT-1 cells after betamethasone treatment ($n \geq 6$) analysed by qRT-PCR. Gene expression was normalized to *Gapdh*. Bars show the mean ± s.d. of the Log2 of Fold Change using basal (complete medium-cultured NIT-1) as standard value (+$p \leq 0.05$ and ++$p < 0.01$, Wilcoxon test).
